# Troll and divide: the language of online polarization

**DOI:** 10.1093/pnasnexus/pgac019

**Published:** 2022-03-10

**Authors:** Almog Simchon, William J Brady, Jay J Van Bavel

**Affiliations:** Department of Psychology, Ben-Gurion University of the Negev, POB 653, Beer Sheva 8410501, Israel; School of Psychological Science, University of Bristol, BS8 1TU, Bristol, UK; Department of Psychology, Yale University, CT 06520-8205, New Haven, CT, USA; Department of Psychology, New York University, New York, NY 10003, USA; Center for Neural Science, New York University, New York, NY, USA

**Keywords:** polarization, trolls, social media

## Abstract

The affective animosity between the political left and right has grown steadily in many countries over the past few years, posing a threat to democratic practices and public health. There is a rising concern over the role that “bad actors” or trolls may play in the polarization of online networks. In this research, we examined the processes by which trolls may sow intergroup conflict through polarized rhetoric. We developed a dictionary to assess online polarization by measuring language associated with communications that display partisan bias in their diffusion. We validated the polarized language dictionary in 4 different contexts and across multiple time periods. The polarization dictionary made out-of-set predictions, generalized to both new political contexts (#BlackLivesMatter) and a different social media platform (Reddit), and predicted partisan differences in public opinion polls about COVID-19. Then we analyzed tweets from a known Russian troll source (*N* = 383,510) and found that their use of polarized language has increased over time. We also compared troll tweets from 3 countries (*N* = 79,833) and found that they all utilize more polarized language than regular Americans (*N* = 1,507,300) and trolls have increased their use of polarized rhetoric over time. We also find that polarized language is associated with greater engagement, but this association only holds for politically engaged users (both trolls and regular users). This research clarifies how trolls leverage polarized language and provides an open-source, simple tool for exploration of polarized communications on social media.

Significance Statement We argue that trolls (including foreign actors) use social media to sow discord among Americans through political polarization. We developed and validated an open-source linguistic tool to gauge polarized discourse on social media and found that 3 distinct troll populations, which hold antiAmerican views, used polarized language more than the average American user. In times of high political instability, misinformation, and disinformation, it is crucial to understand how the enterprise of online foreign interference operates. This research provides insight into the mechanism through which trolls function, and sheds light on the role of language in political warfare. It also provides a dictionary for other scholars to study the online rhetoric of polarization.

## Troll and Divide: The Language of Online Polarization

A growing body of research suggests that the American public has become more polarized over the past few decades (1, 2). These attitudes are mirrored in rising partisan antipathy; dislike toward members of the opposing ideology—a phenomenon known as “affective polarization” (3, 4, 5). The consequences of polarization include growing political radicalism (6), increased ingroup bias (7), and even different behavioral reactions to deadly pandemics (8). The alarming consequences of polarization are by no means limited to America: India, Poland, Columbia, Bangladesh, Israel, Indonesia, Britain, and Brazil, are just some of the many countries facing growing levels of political polarization (9), and some research attributes this intergroup conflict to the rise of social media ((10, 11); but see (12, 13)). In the current paper, we examine the language of online polarization employed by regular citizens and internet trolls.

On social media, polarization is often defined as emerging clusters of like-minded individuals who engage in confirmation bias and curate narratives congruent with their beliefs (14, 15). The formation of like-minded social networks is particularly salient in social media platforms that deploy a news-feed algorithm (e.g. Facebook), or a computational formula that favors some pieces of content over others (16), creating filtered feeds of personalized content (17). The combination of ideological or partisan groups joining like-minded networks and receiving algorithmically determined political content may be amplifying polarization (10, 18, 19, 20). This trend has raised concerns that people may lose a shared sense of reality.

Although recent evidence suggests that general information consumption on social media might not be an echo-chamber for many users (21, 22), there is nevertheless substantial evidence supporting the argument that segregated online communities emerge around politically contentious topics (23, 19, 24, 25, 26, 27, 20). Moreover, exposure to out-group partisans may even increase polarization (28). A damaging effect of ideology-based homophily is enabling and fostering the spread of misinformation (29, 14). Falsehoods appear to spread farther, faster, deeper, and more broadly than the truth on social media, especially for political news (30). As billions of people have opened social media accounts and use these platforms to get their news, it has also exposed them to a hotbed of conspiracy theories, misinformation, and disinformation (31, 32). The rise of misinformation has fueled an international health crisis during the COVID-19 pandemic, leading the World Health Organization to declare this an “infodemic” of misinformation.

There has also been growing concern over the role bad actors may play in online polarization and the spread of misinformation (e.g. antiquarantine messages during COVID-19; (33)). For the past several years, cyberspace has been affected by organized groups of social media users, commonly referred to as “trolls,” who intentionally pollute online discourse. Since 2018, Twitter has been releasing the Twitter Transparency Report, archives of tweets authored by state-affiliated information operations (https://transparency.twitter.com/en/information-operations.html). The most famous of these operations is the Internet Research Agency (IRA), also known as a Russian “Troll Farm,” The IRA has engaged in online political tactics to sew intergroup conflict and influence US citizens during the 2016 presidential election (34) and British citizens prior to the Brexit vote (35). Similarly, other state-affiliated influence operations have been found in numerous countries, including Iran, Bangladesh, Venezuela, China, Saudi Arabia, Ecuador, the United Arab Emirates, Spain, and Egypt (https://transparency.twitter.com/en/information-operations.html). In the current paper, we developed and validated a polarization dictionary and examined whether the rhetoric used by these troll operations was highly polarized.

Some evidence suggests that trolls tend to take on far-right topics and stances, spreading hate speech and islamophobia (36). However, it would be inaccurate to say that trolls are only far-right leaning, and spreading conservative ideology may not even be their ultimate goal. Instead, their main goal appears to be creating polarization and fostering social conflict within democracies. For instance, during #BlackLivesMatter discourse on Twitter Russian trolls were heavily engaged in spreading messages from the 2 ends of the debate; both anti-BLM and pro-BLM (37). The same pattern was observed during online antivaccine debates: trolls were found to echo both positions (pro and against vaccines; (38)). Taken together, these data suggest that online trolls are attempting to polarize social media users during political discourse.

## Overview

The current research had 2 goals: (i) to create a dictionary of polarized language (i.e. linguistic expressions that are associated with political polarization) and (ii) to examine how this language has been used by trolls around the world. We began by building a simple tool to measure polarized language. Previous work studied polarization through network analysis or by exploring topics known to be polarized (39). These methodologies have several advantages (40, 41) but can be computationally expensive, create a barrier for adoption for behavioral scientists who lack the required technical expertise, and are most likely context-dependent which can undercut replicability (42). Here, we sought to validate a dictionary of polarized language that would be applicable across numerous contexts. In what follows, we describe how the dictionary was constructed, its validation using different topics and time periods, and how it tracks dynamic changes in partisan opinions during a time of national polarization (the COVID-19 pandemic).

Next, we examined the online rhetoric of trolls and regular citizens using the polarization dictionary. We conducted a high-powered study using nearly 2,300,000 tweets from trolls in multiple countries and compared the results to a random sample of American Twitter users. To help determine if trolls were using polarized rhetoric more than the average American (38, 43), we examined the levels of polarized language in their tweets when compared to a control group, and explored how levels of polarized language changed over time within each group. These studies suggest that polarized rhetoric was weaponized by online trolls during political discourse.

## Method

### Data collection

We used the SCI lab twitter database at Ben-Gurion University (44). Tweets were collected from all 50 states in the United States and the District of Columbia. We extracted tweets between November 2017 and December 2019. Trolls’ data was taken from the Twitter Transparency Report (45–47). Additional data collection was done using Twitter API 2.0 and the “academictwitteR” R package (48).

All research was conducted in accordance with the Departmental IRB committee at Ben-Gurion University and was ruled “exempt.”

### Preprocessing

Our sample size consisted of 2,306,233 original tweets in the English language (retweets were filtered out): 383,510 by Russian trolls, 329,453 by Iranian trolls, 85,970 by Venezuelan trolls, and 1,507,300 by American Controls (random sample from our Twitter database with no specific text search). Following the exclusion of retweets, English tweets constituted 34% of the Russian trolls dataset, 15% of the Iranian trolls dataset, and 1.25% of the Venezuelan trolls dataset.

For our content-matched analysis, we extracted the 20 most-frequent hashtags that appeared on politically engaged Russian trolls tweets (#MAGA, #tcot, #BlackLivesMatter, #PJNET, #news, #top, #mar, #topl, #Trump, #2A, #IslamKills, #WakeUpAmerica, #FAKENEWS!, #GOPDebate, #NowPlaying, #TCOT, #ccot, #amb, #sports, #TrumpTrain) and searched for tweets posted in the United States with the same hashtags. After the exclusion of retweets, politically engaged Russian trolls sample size was 55,726, and so was their politically matched American controls (55,726).

We could not use our sample of American Controls for Study 4 as it lacked engagement metrics. Therefore, we collected a new control sample, matched in time and without a specific text search (1,144,767).

All tweets had links, tags, and emoticons removed prior to any linguistic analysis. Text mining was done using the "quanteda" package (49) using R (Versions 3.6.3 and 4.0.3).

## Study 1: development and validation of a polarization dictionary

To develop a polarization dictionary, we synthesized data-driven methods and domain expertise. Specifically, we (i) explored the language associated with polarization in a data-driven fashion; (ii) manually pruned the dictionary; (iii) expanded the dictionary by using GloVe word-embeddings (50); and (iv) employed manual trimming. The dictionary contained 205 words (e.g. *corruption, kill, lie, terrorists, political*,and*stupid;* see online materials for the full list) and its full development and psychometric properties are reported in the Supplementary Information. All the materials are publicly available on OSF https://osf.io/bm8uy.

### Dictionary validation

We first validated our dictionary on a subset of the original database used in its construction (19). The database included tweets about contentious political topics that showed a range of ingroup bias in their spread through social networks (i.e. they either were shared with only the political ingroup or spread to 1 or more outgroup members). We built the dictionary on a randomly selected 80% of the original dataset (*N* training set = 19,841) and tested it on the remaining 20% (*N* test set = 5,008). This out-of-sample testing was conducted to ensure the predictive performance of the model and to avoid overfitting. Data preprocessing included removing all duplicates from the data and automatically deleting links and emojis from the text. A polarization score was calculated based on the count of dictionary words in the text, normalized by the tweet length. The means reported below represent the average percentage of the text that was found in the dictionary (for a similar approach see LIWC; (51). Our analysis found that the dictionary successfully discriminated between polarized and nonpolarized tweets from the test set (*M*_polarized _= 6.70, SD _polarized _= 9.08, *N* = 3696 and *M*_nonpolarized _= 4.39, SD _nonpolarized _= 6.60, *N* = 1312), *t* (3156) = 9.79, *P* < 0.001, *Cohen's d’* = 0.27. In other words, our dictionary was able to determine which corpus was more likely to include polarized communications compared to another corpus.

To evaluate generalizability, we validated the polarization dictionary with a different political topic (i.e. different from the original research). We examined the effectiveness of the polarization dictionary in the context of the online #BlackLivesMatter (#BLM) discourse between December 2015 and October 2016, which focused on issues of racial justice in the United States. Prior work had studied the flow of information in #BLM tweets by using a machine learning clustering technique to identify distinct Twitter communities and quantifying the spatial retweet flow within and between clusters (37). The original dataset included 58,698 tweets (https://github.com/leo-gs/ira-reproducibility), and we were able to retrieve 24,747 tweets out of the original sample from Twitter's API. Like in the prior validation, messages were categorized with regard to the spread of information; whether the tweets showed ingroup bias (retweeted within 1 political cluster), or not (retweeted by a user from the other cluster, as classified by the authors). We applied our dictionary to the posts we were able to retrieve, and again we observed that ingroup bias messages contained more polarized language than messages that diffused between clusters (*M*_ingroup bias_ = 5.54, SD _ingroup bias_ = 5.68, *N* = 24,077 and *M*_diffused _= 4.83, SD _diffused _= 5.09, *N* = 670), *t* (716.06) = 3.58, *P* < 0.001, *Cohen's d’* = 0.13. This helped establish the generalizability of our dictionary to a novel political topic.

Beyond testing out-of-sample generalizability, we also tested cross-platform generalizability. We tested the polarization dictionary on the platform *Reddit* using a wider range of political topics. Reddit is an online social media platform that consists of many discussion forums, or communities, called *subreddits*, including several communities devoted to politics (52). We extracted up to 1,000 messages from 36 political communities with established ideologies (18 from each political side). As a control group, we sampled up to 1,000 messages from 18 other communities, randomly sampled from a list of popular subreddits (https://github.com/saiarcot895/reddit-visualizations). We collected 53,859 posts between June 2015 and December 2018 from the Pushshift Reddit API (53). Following data cleaning, our sample size consisted of 49,230 original posts. We applied the polarization dictionary on the Reddit sample and conducted a one-way between-group ANOVA. A planned comparison between the political groups revealed a significant difference between the control and the other political communities (*M*_left_ = 2.38, SD _left_ = 4.61, *N* = 17,005; *M*_right _= 2.57, SD _right _= 5.34, *N* = 15,859; and *M*_control _= 0.97, SD _control _= 3.44, *N* = 16,366), *t*(49,227) = 34.81, *P* < 0.001, *Cohen's d’* = 0.31. More information is reported in the Supplementary Information. In other words, the rhetoric in political Reddit groups was more polarized than apolitical Reddit groups.

As a more stringent sensitivity test, we replaced the randomly sampled control group with a “neutral” reference of contentious topics. We extracted messages from the popular subreddit *NeutralPolitics* (www.reddit.com/r/NeutralPolitics), a reddit community devoted to factual and respectful political discourse. This sample consisted of 9,984 posts between April 2016 and December 2018 (9,772 after data cleaning). A planned comparison between the political groups revealed a significant difference in polarized rhetoric between *NeutralPolitics* and the other political communities (*M*_left_ = 2.38, SD _left_ = 4.61, *N* = 17,005; *M*_right _= 2.57, SD _right _= 5.34, *N* = 15,859; and *M*_neutral _= 2.24, SD _neutral _= 4.49, *N* = 9,772), *t*(42,633) = 4.12, *P* < 0.001, *Cohen's d’* = 0.04. See Supplementary Information for more details. This suggests that polarized rhetoric was reduced among the reddit community focused on respectful political discourse (although we note that the effect size here is very small).

To determine if our dictionary would track dynamic changes in polarized public opinions over time, we compared polarized language with polls about US citizens’ concern about the COVID-19 pandemic. The data were collected from a representative panel by Civiqs (https://civiqs.com/results/coronavirus_concern), an online polling and analytics company. Recent polls have revealed clear partisan differences between Democrats and Republicans in reported concerns about the COVID-19 pandemic—such that Democrats are consistently more concerned about the pandemic than Republicans (54). We tested whether the language in tweets about coronavirus was associated with the partisan discrepancy in public opinion about COVID-19. We calculated a “partisan difference score” from 2020 February 25th until 2020 April 14th by subtracting the daily Republican net concern from the daily Democratic net concern, as reported by Civiqs (the specific question was “how concerned are you about a coronavirus outbreak in your local area?”). The poll was based on responses from 22,256 respondents and included measures to avoid demographic and ideological biases.

**Figure 1.**
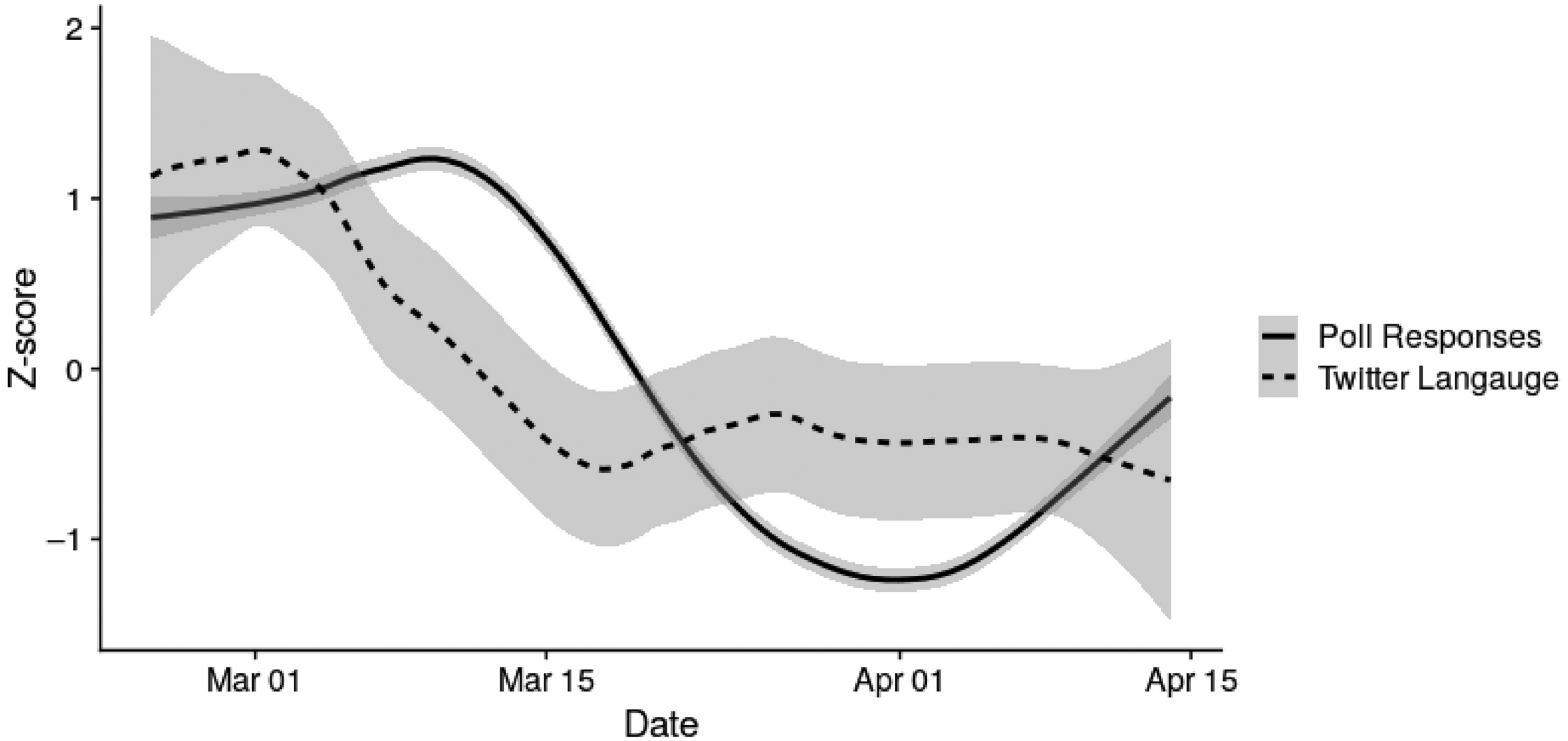
Dynamic polarization changes in polls of COVID-19 concern and polarized language on Twitter. The solid line represents partisan differences in COVID-19 concern (*N* = 22,256), and the dashed line represents the degree of polarized discourse on Twitter (*N* = 553,876, dashed line). Values on the *X*-axis represent the time, and values on the *Y*-axis represent standardized scores of the variables. The functions have gone through a locally estimated scatterplot smoothing (span = 0.33, degree = 1). Shaded areas around the regression line denote 95% CI.

To compare Twitter language to partisans’ concern, we collected 553,876 Twitter messages from the United States within these dates that used the terms “covid” or “coronavirus.” We then applied the polarization dictionary to the tweets and aggregated by date. We found that polarized language on social media, measured by the mean % of words from our dictionary contained in the tweets, was positively associated with partisan differences in concern about the COVID-19 pandemic over time, *r* (48) = 0.45, *P* = 0.001, see Figure 1. A post hoc analysis revealed that the correlation between poll responses and twitter language was strongest when Twitter language was lagged by 8 days (i.e. poll_t0_, twitter_t8_) *r* (40) = 0.67, *P* < 0.001 (for a full lag table of 16 days, see Table S1, Supplementary Material). In other words, polarized rhetoric about COVID-19 mirrored polarization in public opinion over the early phase of the pandemic. This also suggests that the polarization dictionary may be useful in detecting future patterns of public opinion.

Taken together, these 4 sets of analyses (cross-validation, out of set validation, cross-platform validation, and predictive validation) provide converging validity for the dictionary, showcasing its ability to capture political polarization in language across 4 different contexts. For a summary of all validation steps, see Table 1.

**Table 1.** Summary of validation steps. Effect sizes correspond to Cohen's *d’* or Pearson's *r*. All tests are significant at *P* < 0.001.

Validation type	*N*	Effect size
Cross-validation	5,008	*d* = 0.27
Out of set (BLM)	24,747	*d* = 0.13
Cross platform (Reddit)	49,230	*d* = 0.31
Predictive validation (COVID)	553,876	*r* = 0.45

## Study 2

### Study 2a: polarization in Russian trolls

Russian trolls, or anonymous social media accounts that are affiliated with the Russian government, were active around highly contentious political topics around the world, including in the United States and Britain (34, 35). With the release of the Twitter Transparency Report, a sample of the Russian and other countries’ operations were officially disclosed and used to study the role of trolls in amplifying political polarization (37, 38, 55). Therefore, we hypothesized that state-affiliated trolls would use more polarized language on social media compared to ordinary Twitter users. We also examined how polarized language may have changed over time. For instance, if trolls' levels of polarized language are increasing over time, it would imply that trolls are spending increased energy toward tactics that sow discontent and aim to influence polarized discourse. On the other hand, levels of polarized language might be increasing among American Twitter users as well, similar to trends of affective polarization (5).

#### Results

We compared Twitter messages posted by trolls to an American control sample (collected from across the United States through the Twitter API). We only used original tweets that were posted in the English language and were most likely aimed for an international/American audience. The comparison was matched for the same time range (2016 November 23–2018 May 30). We applied the polarization dictionary, which was generated from and validated on different datasets (see Study 1) to extract polarization scores. First, we found that Russian trolls (*M* = 2.37, SD = 5.14, and *N* = 61,413) used significantly more polarized language than tweets sent by the control sample (*M* = 1.47, SD = 5.35, and *N* = 516,525), *t*(78,081) = 40.96, *P* < 0.001, and *Cohen's d* = 0.17. These results suggest that trolls are leveraging polarized language to push conflict among the US citizens in the context of political discourse. For the top 25 most used words adjusting for their frequency (tf-idf), see Figure S1 (Supplementary Material).

However, not all trolls are equal. Research suggests that Russian trolls could be classified into 5 distinct types: Right, Left, News, Hashtag Gamers, and Fearmongers (56). It could be argued that a cleaner analysis would only constitute Left and Right trolls, and should be contrasted with a politically engaged American sample. Therefore, we used the Russian Troll classification (Shared in partnership with FiveThirtyEight on https://github.com/fivethirtyeight/russian-troll-tweets) (56), and matched an American sample for their content (via hashtag use, see Method section), posting time (January 2015–May 2018) and quantity. Again, we find that politically oriented Russian trolls use significantly more polarized language than their politically matched American sample (Russian trolls: *M* = 5.16, SD = 8.00, and *N* = 55,726; American controls: *M* = 2.91, SD = 6.84, and *N* = 55,726), *t*(108,836) = 50.61, *P* < 0.001, and *Cohen's d* = 0.30 (for a robustness check, see Supplementary Materials).

**Figure 2.**
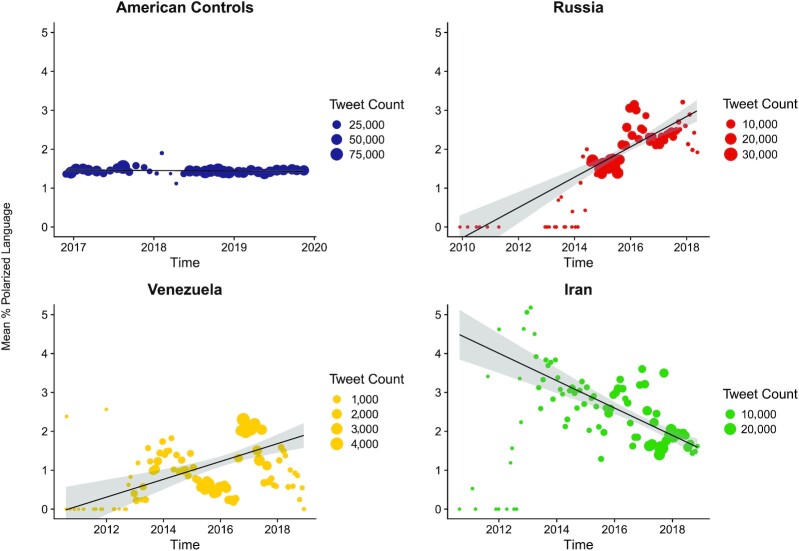
Scatter plot of the average polarized score by Twitter sample. We examined monthly polarized language in American controls (*N* = 1,507,300; blue), and trolls from Russia (*N* = 383,510; red), Venezuela (*N* = 85,970; yellow), and Iran (*N* = 329,453; green). Values on the *Y*-axis represent the average % of polarized language in the month. The size of the dots corresponds to the monthly sample size. Shaded areas around the regression line denote 95% CI. Note that the *Y*-axis is fixed to 0–5, data points exceeding this limit are not shown in the figure; the regression lines take these observations into account. Results indicate that trolls from Russia and Venezuela have been increasing their use of polarized rhetoric, but Americans have not.

To determine if polarized language is increasing over time, we sampled 1,507,300 tweets that were posted between November 2016 and December 2019 in the United States. These tweets were pulled randomly from BGU's SCI lab twitter database (sampling approach described in the Method section), with no specific text search. We applied the polarization dictionary to the text and aggregated by months. We conducted a weighted linear regression with monthly observations as the weighting factor. We found that Russian trolls used far more polarized language as time progressed (*b* = 0.03), *R^2^ = 0*.46, *F*(1, 69) = 58.85, and *P* < 0.001, Moreover, this was a strikingly large effect size. We did not find the same pattern among American control users, (*b* = −0.001) *R^2^ = 0*.05, *F*(1, 35) = 1.90, and *P* = 0.178 (see Figure 2). This suggests that trolls are increasing the use of polarized language much faster than ordinary users, independent groups correlation comparison *z* = 5.06, 95% CI [.55, 1.21], and *P *< 0.001.

This finding suggests Russian trolls have increased their use of polarized rhetoric, but the average US Twitter does not show evidence of mirroring the type of language used by the trolls. This could be because trolls are only reaching and influencing the most politically active Twitter users, or that the average user expresses polarized attitudes in different ways. However, we note that the time frame for trolls and controls is not identical. As such, any differences in these trends should be treated as tentative. That said, in a post hoc analysis conducted on the same time frame (2016 November 23–2018 May 30), Again, Russian trolls used far more polarized language as time progressed (*b* = 0.03), *R^2^ = 0*.51, *F*(1, 17) = 17.48, and *P* < 0.001 while American control users did not, (*b* = 0.006), *R^2^ = 0*.16, *F*(1, 17) = 3.14, and *P* = 0.094 (however note the small sample sizes in this analysis).

### Study 2b: polarization in Venezuelan and Iranian trolls

We, next, sought to see if this pattern of polarized language generalized to other political contexts and countries. Given Russia's effort at online political warfare (57), we also tested whether polarization attempts extended to other political actors. Russia, Iran, and Venezuela all hold antiAmerican views and share warm relationships with each other (58, 59, 60). Therefore, these countries may have incentives to meddle with American politics. We analyzed trolls from these nations to see if they were using similar polarized rhetoric to sow conflict with Americans.

#### Results

We compared Twitter messages posted by Venezuelan and Iranian trolls (identified by Twitter^1^) to a neutral American control sample. Again, we only used original tweets that were posted in the English language which were most likely aimed for an international/American audience. The paired comparisons were again matched for the same time range. In both the countries we examined, the tweets sent by trolls used significantly more polarized language than tweets sent by American control samples (*P*s < 0.001), see Table 2. For the top 25 most-used words adjusting for their frequency (tf-idf), see Figure S1 (Supplementary Material).

**Table 2.** Means, SDs, sample sizes, and time range for each troll group comparison with American controls. The table consists of *t* statistics, degrees of freedom, and *Cohen's d’*. All the *t* tests are significant at *P* < 0.001.

	Trolls		American controls					
	Mean (SD)	*N*	Mean (SD)	*N*	Date range	*t*	*df*	*Cohen's d*
Iran	2.15 (4.66)	220,628	1.46 (5.26)	929,908	2016–11–23–2018–11–28	61.32	366,496	0.13
Venezuela	1.80 (4.67)	30,987	1.45 (5.25)	953,197	2016–11–23–2018–12–07	12.87	33,588	0.07

Following the same analysis as in Study 2a, we conducted a weighted linear regression with monthly observations as the weighting factor, and found a diverging pattern between populations of trolls: Whereas trolls based in Venezuela used more polarized language as time progressed (*b* = 0.02), *R^2^ = 0*.19, *F*(1, 91) = 20.91, and*P* < 0.001, Iranian trolls used less polarized language (*b* = -0.03), *R^2^ = 0*.33, *F*(1, 85) = 41.06, and *P* < 0.001, see Figure 2. Therefore, any trends in polarized language might be specific to the foreign nation involved.

## Study 3: exploratory topics of polarization

In Study 2, we showed that foreign agents from various countries strategically used polarized language in social media communications, and in a majority of cases we see an increase over time in these attempts. One remaining question is what specific forms of polarized rhetoric are leveraged by different groups. Investigating the themes associated with polarized language could shed light on the social–psychological processes capitalized by trolls, and generate better intuition on their strategies. Therefore, we conducted an exploratory analysis in which we decomposed the dictionary into different factors to determine whether these factors could contribute to our understanding of the trends in online polarized rhetoric.

**Figure 3.**
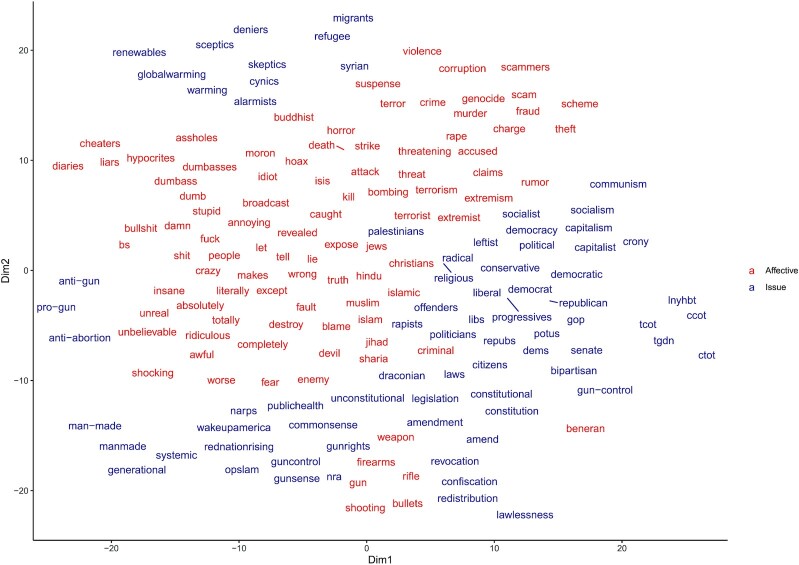
Semantic space representation of the Polarization Dictionary. *X*- and *Y*-axes represent t-SNE dimensionality reduction of GloVe embeddings. Words in red mark the first cluster (“Affective”) and words in blue mark the second cluster (“Issue”).

As in Study 1, we used GloVe word embeddings as a high-dimensional representation of the words in the polarized dictionary (50) and conducted hierarchical clustering analysis. We observed that in the highest level of division, the algorithm clustered the words in a manner that is somewhat consistent with the theoretical separation between *issue* and *affective* polarization (see Figure 3 and Figure S2, Supplementary Material). See Supplementary Information for more information.

Scholars have made the conceptual distinction between issue polarization—an ideological, policy-based political divide, and affective polarization, i.e. dislike, distrust, and general animosity of political partisans toward the other political side (5, 61, 62). This distinction is roughly reflected in the dictionary. For example, while the Issue subcomponent addresses ideological and policy keywords (e.g. *liberal*, *conservative*, *socialism*, and *gun-control*), the Affective component references instances of negative moral–emotional words (e.g. *kill*, *destroy*, and *cheaters*; (19)), and distinct ethnic and religious groups (e.g. *Muslim* and*Jews*). We should note that this is our own interpretation of the clusters and other theoretical mappings may fit as well (e.g. Affective could be interpreted as partisan taunting (63); therefore, the labels Affective and Issue should be treated as relatively fuzzy concepts.

With these divisions in mind, we tested whether language associated with issue vs. affective polarization was associated with differential language use among trolls and ordinary users.

### Results

We applied the 2 subsets of the polarization dictionary on the social media messages posted by trolls and a random sample of American users. As in Study 2, we compared polarization levels between the groups (paired comparisons matched for the same time range). In all the countries we examined, the tweets sent by trolls used significantly more polarized language than tweets sent by American control samples (*P*s < 0.005), both on affective and issue polarization, see Table 3 and Figure S3 (Supplementary Material). Temporal analyses are reported in the Supplementary Information.

**Table 3.** Means, SDs, sample sizes, and time range for each troll group comparison with American controls by Issue and Affective polarization components. The table consists of *t* statistics, degrees of freedom, and *Cohen's d’*. All the *t* tests are significant at *P* < 0.005.

	Trolls		American control					
	Mean (SD)	*N*	Mean (SD)	*N*	Date range	*t*	*df*	*Cohen's d*
Issue polarization								
Russia	0.51 (2.35)	61,413	0.10 (1.00)	516,525	2016–11–23–2018–05–30	42.68	64,104	0.34
Iran	0.33 (1.70)	220,628	0.11 (1.08)	929,908	2016–11–23–2018–11–28	58.55	264,182	0.18
Venezuela	0.39 (1.99)	30,987	0.11 (1.07)	953,197	2016–11–23–2018–12–07	24.59	31,575	0.25
Affective polarization								
Russia	1.86 (4.62)	61,413	1.37 (5.24)	516,525	2016–11–23–2018–05–30	24.53	81,500	0.09
Iran	1.82 (4.29)	220,628	1.34 (5.14)	929,908	2016–11–23–2018–11–28	44.69	386,294	0.09
Venezuela	1.41 (4.19)	30,987	1.34 (5.14)	953,197	2016–11–23–2018–12–07	2.84	34,087	0.01

In the current exploratory study, we showed that the polarization dictionary is composed of 2 subcomponents that map onto theoretical elements of polarization (Issue and Affective). In addition, we showed that all troll groups use more polarized language than a random sample of American social media users and that this holds for both affective and issue polarization (although effect sizes of issue polarization are substantially larger).

## Study 4: polarized language and engagement

Studies 2 and 3 demonstrated a link between polarized language use and troll accounts on social media. Indeed, our findings are consistent with the idea that trolls sow discord among Americans by using polarized language in conversations with others. Yet our results are agnostic to whether polarized language creates divisions vs. merely reflects an existing polarized state. To address this ambiguity, in the current study we investigate the extent to which polarized language is associated with increased engagement If polarized language used by trolls draws more engagement from ordinary social media users, it would demonstrate that when trolls seed polarized language online, users become active agents in spreading the polarized messaging among groups.

Engagement of polarized language is an important metric because engagement with political content online is generally associated with high levels of ingroup bias; that is, it is far more likely to be shared within the political ingroup than in the outgroup (18, 19, 16). For example, 1 of the key predictors of engagement on Twitter and Facebook in the political context is outgroup animosity (64). Whether intentional or not, if polarized language used by Trolls is associated with increased engagement, it would suggest that Trolls’ language use has potential to exacerbate division among political users (even if users were already divided).

### Results

**Figure 4.**
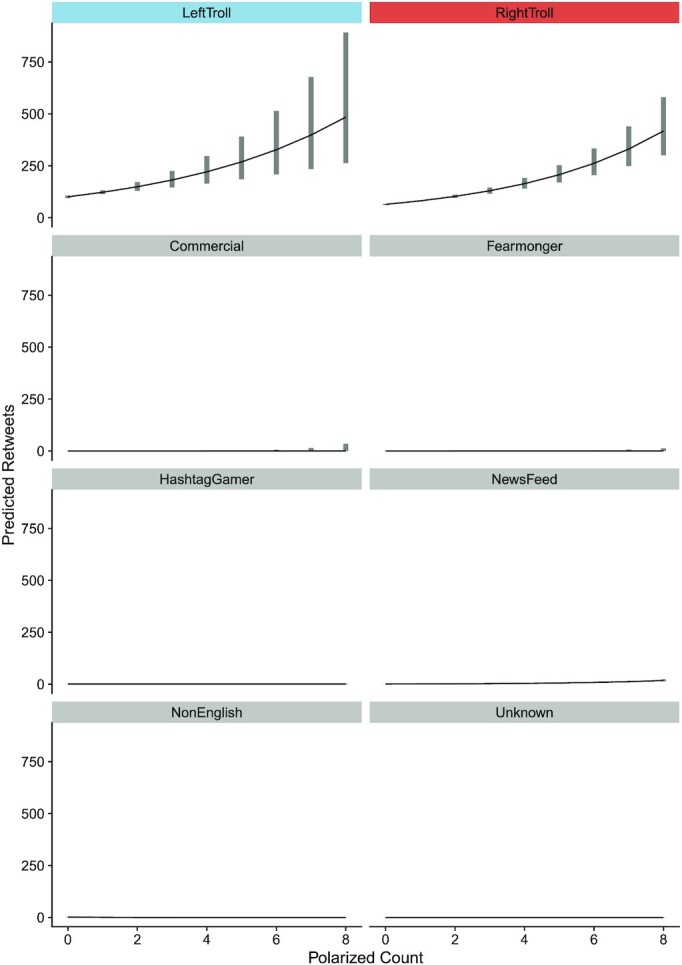
Polarized language predicts retweets in political Russian trolls. The graph depicts the number of retweets predicted for a given tweet as a function of polarized language present in the tweet and type of troll. Bands reflect 95% CIs. For varying *Y*-axes, see Figure S5 (Supplementary Material).

To test whether polarized language is associated with selective engagement in political discourse, we utilized the Russian Trolls Classification data (56) from Study 2. We only included observations for which we had an engagement metric (i.e. retweet count, *N* = 118,215). For ease of interpretation, we counted the number of words in the dictionary and used it to predict the retweet count in a negative binomial generalized linear model. We find that for every polarized word in a tweet, retweets increase by 72%, IRR = 1.72, CI [1.64, 1.79], and *P* < 0.0001. Next, we added an interaction by troll category. We conducted an Analysis of Deviance and found both main effects and interaction term significant (Polarized Language: *χ^2^*(1) = 5263*, P *< 0.0001; Troll Category: *χ^2^*(7) = 373,563*, P *< 0.0001; and Polarized Language * Troll Category: Category: *χ^2^*(7) = 2223*, P *< 0.0001). In a planned comparison we find that this effect stems directly from political trolls, Political vs. non Political trolls ratio = exp(1.35*10^15^), 95% CI [exp(5.03*10^14^), exp(2.2*10^15^)], and *P *< 0.0001, see Figure 4.

We take these results as evidence that polarized language is indeed polarizing, however, there is no reason to assume this effect applies strictly to trolls. We conducted the same analysis on samples of politically engaged controls (Study 2, *N* = 55,726), and a new sample of American controls for which we obtained engagement metrics (*N* = 1,144,767). Again, we find that in the politically engaged controls there is a positive association between polarized language and retweets, such that for every polarized word in a tweet, retweets increase by 39%, IRR = 1.39, 95% CI [1.35, 1.48], and *P* < 0.0001. However, in a random sample of Americans we do not find a significant association IRR = 1.19, 95% CI [0.80,1.77], and *P* = 0.390.

We should note that these analyses are usually done with the number of followers as a covariate, yet retrospective information was only available for the trolls’ dataset. For transparency, we show here the analysis controlling for the covariate. After adding followership in the trolls analysis we find the same pattern of results, however the effect size diminishes: IRR = 1.61, 95% CI [1.57, 1.67], and *P* < 0.0001; planned contrasts: Political vs. non Political trolls ratio = exp(7.6*10^9^), CI [exp(3.6*10^9^), exp(1.21*10^10^)], and *P *< 0.0001.

Overall, these results indicate that polarized language is associated with greater traction on social media, but only in political contexts. Since the probability of a political message to be retweeted within the political ingroup is far greater than the outgroup, we take this as evidence that polarized language is not only a marker for a static polarized state, but contributes to the polarization process.

## Discussion

We developed and validated a dictionary of polarized language used on social media. We validated this dictionary using 4 strategies and showed it consistently detected polarized discourse on Twitter and Reddit on multiple topics and corresponded well to the dynamics of partisan differences in attitudes towards the COVID-19 pandemic. We found that state-affiliated trolls from Russia and other countries use more polarized language than a random sample of American users and that while the language of Russian and Venezuelan trolls have used more polarized rhetoric with time, levels of polarized language in American controls did not increase. We found that our data-driven dictionary taps into distinct theoretical elements of polarization, and that trolls from all tested countries use more polarized rhetoric in both issue and affective factors (broadly denied). Lastly, we showed that polarized language is associated with more traction on social media, but only in political contexts; this finding suggests that polarized language advances polarization and not merely reflects it.

These results expand on prior work documenting trolls’ attempts to pollute the online environment with polarized content and sow discord among Americans (65). We provide novel evidence that this mission spans several countries that hold antiAmerican views. Prior research has revealed that when exploring the clusters of polarized topics, trolls are often found in the centroids of these clusters, driving the partisan discourse on both ends (37, 38, 55). Our research extends these findings; we found that trolls share controversial content and engage in highly polarized issues, but that they also use higher levels of polarized language as a tool in their discourse. In addition, we found that polarized language is associated with greater engagement, however, this association only holds for politically engaged users—both trolls and controls. This is consistent with a view that trolls’ use of polarized language is intended and weaponized in order to sow polarization, however our methods are not sufficient to draw such causality.

Questions remain as to the extent of influence of trolls’ social media presence on real people. However, it is important to note that even a small number of agents with aggressive attitudes can have a substantial influence on the majority view, a process called “information gerrymandering” (66). Exposure to polarizing attitudes even produced by a small number of agents can have a devastating effect on political compromise in a social network; such findings suggest that trolls have the ability to influence many of the users on social networks. Furthermore, recent evidence suggests that troll's messages propagate to mainstream media and are represented as “the voice of the people” (67). This way, trolls win twice: once when they share the polarized content, and then again when it is being echoed on other media platforms, creating a polarizing loop.

However, some are skeptical of the change trolls may impose on people's attitudes. A recent paper followed over 1,200 American Twitter users for the course of 1 month in late 2017. The authors found that only a small fraction of users interacted with Russian trolls, and they did not observe any change in partisan attitude during that time among these users (68). In a study that explored the domestic effect of Russian trolls (i.e. messages that were targeted inwards to Russian users), it was found that trolls were trying to promote a progovernment agenda and dissolve government criticism (69); nevertheless, trolls were only successful at the latter, suggesting their influence is restricted in scope. While our results cannot speak to causal factors, we do find that while levels of polarized language were rising in Russian trolls, this was not the case among American users. Future research is required to understand the precise impact trolls have in reference to specific political events.

Given the evidence on the growing polarization and partisan antipathy in the American public (5), we also explored whether polarized discourse on social media would increase with time among a sample of American users. We did not find evidence to support this hypothesis; levels of polarization did not increase across time, suggesting that polarized discourse among average American users did not grow between November 2016 and December 2019. These results are consistent with other findings that do not find evidence for increased polarization during this brief time frame (70). This could suggest that polarized discourse has not changed, that it has reached a plateau, or that American users’ way of expressing polarized language has changed slightly over time. Discerning between these possibilities is an important endeavor for future research.

This paper also introduced the polarization dictionary and showcases its validation and application in studying political polarization. The dictionary is easy to use and can be utilized externally with LIWC (51), or with the example code provided in the Supplementary Information for *R*. Having a quantifiable measure of polarized language in social media messages is a quick way to estimate polarization levels that aligns with other current practices, wherein researchers relied on computationally extensive network analyses, or narrowed down to a specific partisan topic to carry out their studies.

The current study has several limitations. The polarization dictionary has been built on data collected in 2015 and on 3 polarized topics. Therefore, it is subjected to bias about topics that were timely in 2015 and is potentially restricted in its scope. We attempt to get around this limitation by expanding the lexicon using word-embeddings and testing its validation over multiple time periods. Nonetheless, language is highly dynamic on social media and our dictionary should always be validated when applied to a new context. Given its data-driven development, it also includes some terms that may not seem strictly polarized (e.g. *people*). Therefore, if being used by other researchers, we recommend using it comparatively by having a baseline corpus and measuring amounts of polarized language between groups to get a relative estimate.

A potential issue is with the authenticity of early social media accounts identified as trolls. Some countries use hacked, purchased, or stolen accounts. Early data, therefore, may not have originated with the nation in question. While this was probably not the case with the Russian trolls dataset, it could be the case with some Venezuelan or Iranian content, and may have biased our polarization over time analyses. That said, we employed a weighted regressions analysis that takes into account the relatively sparse nature of early messages (and therefore, downweights their importance). These analyses complement the Russian sample and provide a wider, descriptive view of how different troll populations use polarized language.

In addition, this work has focused primarily on quasi-experimental manipulations or correlational methodology. Future work should examine if there are causal factors that increase or decrease polarization. For instance, given the potential influence that the design of social media can have on moralized language (29), it is possible that specific design feature changes could impact polarization language. For instance, down-weighting polarized language on social media news feeds might influence attitudes such as partisan antipathy.

## Conclusion

Taken together, this research offers a tool to detect and understand the use of polarized rhetoric on social media. In times when it seems like we have reached toxic levels of polarization in America, it is increasingly important to continually develop tools to study and combat the potentially polarizing influence of foreign agents in American politics.

## Funding

This work was partially supported by research grants through Grant number 61378 from the John Templeton Foundation to J.V.B.

## Supplementary Material

pgac019_Supplemental_File
