# Supplementary material for: Troll and divide: the language of online polarization
Source: PNAS Nexus. 2022 Mar 10;1(1):pgac019. doi: 10.1093/pnasnexus/pgac019 (PMC9802075; doi:10.1093/pnasnexus/pgac019)
Supplement: pgac019_Supplemental_File [file pgac019_supplemental_file.pdf]

Supplementary Information for

Troll and Divide: The Language of Online Polarization

This document includes:

Supplementary Text
Tables. S1, S2
Figures. S1- S5
References

For the dictionary, full code and analysis see OSF repository: https://osf.io/bm8uy

Study 1: Development of a Polarization Dictionary

Dictionary Development

We built on the work by Brady et al. (2017) which studied the diffusion of controversial political content on Twitter during discussions of climate change, same-sex marriage, and gun control. Their dataset included 24,849 tweets with available information on whether the tweets were polarized (retweeted within one political community), or not (retweeted by a user from the opposing ideology). We performed a differential language analysis, a procedure in which two groups are compared in their frequency of word use (Schwartz et al., 2013), on 80% of their data (the rest was kept for validation). We compared the word use of the polarized cluster vs. the non-polarized cluster by calculating a chi-square statistic for every word in the data set; resulting in a shortlist of words that were significantly associated with polarization.

In the second step, we manually pruned the list of words (i.e., the dictionary) by filtering out names of individuals (e.g. *Bernie*) and topical words (e.g., *antarctic)* that would be unlikely to generalize to other contexts outside the original research (Brady et al., 2017). The full dictionary (N = 256) was judged independently for pruning by two of the authors (A.S. and W.J.B.) and agreement reached a Cohen's κ of 0.61, z = 9.93, p <. 001, 95% CI [.51, .72]. Remaining disagreements were discussed to reach convergence. Words that were associated with depolarization were removed. The pruned version of the dictionary consisted of 57 words.

Next, we used word embeddings, a vectorized representation of words that encompasses semantic fields, to expand the pruned dictionary. This process helped the dictionary capture a greater linguistic space while staying close to the semantic space implied from the dictionary. The GloVe algorithm (Pennington et al., 2014) utilizes word co-occurrence in large corpora to create embeddings of 200 dimensions. We used a pre-trained GloVe model by Stanford NLP which was built on 2 billion tweets (https://github.com/stanfordnlp/GloVe) to extract the five most semantically-related words to each of the "seed" words from the prior step. For example, the word *threat* was expanded by the words *threats, attacks, terrorism, targets,* and *threatening*. The fully expanded dictionary contained 232 words.

In the final step, we trimmed proper names (e.g., *Obama*) and nonsensical additions (e.g., *prettylittleliars*). This time there was perfect agreement between the raters in applying the two rules, which resulted in the removal of 27 words (the final dictionary contained 205 words; the word lists with raters agreement are found on the Online Repository).

Internal Consistency

Conducting psychometric assessments of dictionaries is a well-known issue in text analysis (Pennebaker et al., 2007). Especially in the context of social media and even more so when using Twitter data, it is important to understand what is the unit of analysis in the psychometric evaluation. To conduct an analysis of internal consistency, we grouped together tweets of the same authors. Originally our training set consisted of 19,841 tweets. After grouping tweets together by authors, the training corpus consisted of 7,963 observations. To assess internal consistency in the *binary method* (Pennebaker et al., 2007), we calculated a binary occurrence matrix of the dictionary elements wherein each word in the dictionary is considered an item in the "questionnaire" (i.e., the dictionary), and calculated Cronbach's *alpha* of 0.75, 95% CI [0.75,0.76].

Dictionary Validation

Reddit Analysis

We extracted reddit comments from 36 politically mapped subreddit (Soliman et al., 2019). The list of subreddits and their political orientation is shown in Table S2.

Since many comments on Reddit do not contain more than a title, we combined the title and the body of the message into a unified text variable. We then removed links and emoticons and filtered out deleted or removed messages. Messages in languages other than English were removed as well. Reddit messages were collected through the Pushshift API and using the *rreddit* R package (Kearney, 2019).

Results. We applied the dictionary on the Reddit sample (political left, political right and control group) and conducted a one-way between-group ANOVA. Results show a significant effect of political group $F(2,49227) = 610.65$, $p < .001$, $\eta_p^2 = .024$, which was followed by a planned comparison reported in the main text. The second analysis included a neutral sample (*NeutralPolitics*) instead of control messages collected from a random sample of popular communities. We applied a one-way between-group ANOVA. As before, results show a significant effect of political group $F(2,42633) = 14.51$, $p < .001$, $\eta_p^2 < .001$, which was followed by a planned comparison reported in the main text.

Studies 2 and 3: Term frequency-inverse document frequency analysis

To better understand the type of language that drives differences in polarized language between trolls and American controls, we conducted a term frequency-inverse document frequency (tf-idf) analysis; a statistical procedure that marks the word importance in a corpus, based on comparing a word's frequency with its base-rate usage. We selected only the words that appear in the polarization dictionary and ranked them by their tf-idf value. Figure. S1 displays the top 25 polarized words based on their tf-idf value.

Results

We used the Russian Troll classification (Linvill & Warren, 2020), and matched an American sample for their content (via hashtag use, see Method section), posting time (January 2015 - May 2018) and quantity. A closer look reveals that one of the popular hashtags which was used for content-matching may not be a good sampling decision (#NowPlaying). Therefore, as a robustness check we decided to analyze the data with the exclusion of tweets containing this hashtag. The results continue to show more polarized language in politically-oriented Russian trolls vs. politically-matched Americans $t(103{,}528) = 39.48$, $p < .001$, *Cohen's d* = 0.24.

Study 4: Exploratory Topics of Polarization

Hierarchical Clustering

To conduct a thematic clustering of the polarization dictionary, we extracted GloVe word embeddings (Pennington et al., 2014). We then lemmatized the words in the dictionary, and for every word that shared a lemma, we took the average embedding of that lemma, resulting in 170 words in total for clustering. Next, we conducted hierarchical clustering analysis and cut the clustered at the highest level of division (2). See dendrogram in Figure S2.

Results

We applied the two subsets of the polarization dictionary on the social media messages posted by trolls and a random sample of American users across time. As in Studies 2 and 3, we calculated monthly polarization scores and conducted a weighted linear regression predicting polarized language as a function of time, dictionary subcomponent and their interaction with monthly observations as the weighting factor. We were interested in whether the slope of the two

dictionary components differ in each group. While there were no significant interactions in the Russian or Venezuelean groups, we found that in American controls, issue polarization had a positive slope, however not significant *b* = 0.0004, *SE* = 0.0005, 95% CI [-0.0005,0.0016], while affective polarization had a significant negative slope *b* = -0.001, *SE* = 0.0005, 95% CI [-0.0024,-0.0003], resulting in a significant slope difference *b* = -0.001, *SE* = 0.0072, $t(70) = -2.55$ $p = .013$. We also found that in Iranian trolls, both affective *b* = -0.0207, *SE* = 0.0026, 95% CI [-0.0259,-0.0155] and issue polarization *b* = -0.0059, *SE* = 0.0026, 95% CI [-0.0111,-0.0007] had significant negative slopes, which differ significantly from each other *b* = 0.0148, *SE* = 0.0036, $t(170) = -4.015$ $p < .001$, see Figure S3.

In the current exploratory study, we showed that the polarization dictionary is composed of different subcomponents that map onto theoretical elements of polarization. In addition, we show that the lack of significant polarization trend in American controls, could be attributed to the different trends in affective and issue polarization. On a closer look, affective polarization showed a significant negative trend, however further inspection revealed the trend is driven by a relatively high value which was given the most weight, namely August 2017. When omitted from the analysis, the negative trend was no longer significant *b* = -0.001, *SE* = 0.0005, 95% CI [-0.0017, 0.0001].

Interestingly, in August 2017 the United States had experienced one of most contentious events in its recent history. “Unite the Right” rally in Charlottesville, Virginia was an exemplar of a hyper-polarized event, resulting in a white supremacist killing one person and injuring 19 other people (Tien et al., 2020). Therefore, while contributing to a potentially inaccurate trend, high levels of affective polarization in August 2017 do make sense given the context.

Table S1. Correlation table between poll responses and lagged twitter language. Adjusted for multiple comparisons using the Holm method.

Lag	*r*	CI low	CI high	*t*	*df*	*p*
1	0.47	0.21	0.66	3.62	47	0.076
2	0.48	0.23	0.67	3.75	46	0.054
3	0.50	0.25	0.69	3.89	45	0.036
4	0.52	0.28	0.71	4.08	44	0.021
5	0.56	0.32	0.73	4.45	43	0.007
6	0.61	0.38	0.77	4.99	42	0.001
7	0.66	0.44	0.80	5.57	41	<.001
8	0.67	0.46	0.81	5.70	40	<.001
9	0.67	0.46	0.81	5.63	39	<.001
10	0.66	0.44	0.81	5.47	38	<.001
11	0.64	0.41	0.79	5.07	37	0.001
12	0.62	0.38	0.79	4.78	36	0.003
13	0.61	0.36	0.78	4.55	35	0.007
14	0.60	0.34	0.77	4.36	34	0.013
15	0.58	0.30	0.76	4.06	33	0.032
16	0.54	0.25	0.74	3.63	32	0.104

Table S2. List of known politically leaning subreddits, adapted from Soliman et al. (2019).

Subreddit	Political Leaning
BlueMidterm2018	Left
CaliforniaForSanders	Left
DepthHub	Left
Enough_Sanders_Spam	Left
esist	Left
FriendsofthePod	Left
GrassrootsSelect	Left
GreenParty	Left
justicedemocrats	Left
Keep_Track	Left
Kossacks_for_Sanders	Left
LateShow	Left
Maher	Left
occupywallstreet	Left
Political_Revolution	Left
PoliticalDiscussion	Left
progressive	Left
RussiaLago	Left
altright	Right
AskThe_Donald	Right
CBTS_Stream	Right
DarkEnlightenment	Right
DrainTheSwamp	Right
europeannationalism	Right
greatawakening	Right
hottiesfortrump	Right
kekistan	Right
Le_Pen	Right
Mr_Trump	Right
Physical_Removal	Right
redacted	Right
The_Congress	Right
The_Europe	Right
The_Farage	Right
tucker_carlson	Right
WhiteRights	Right

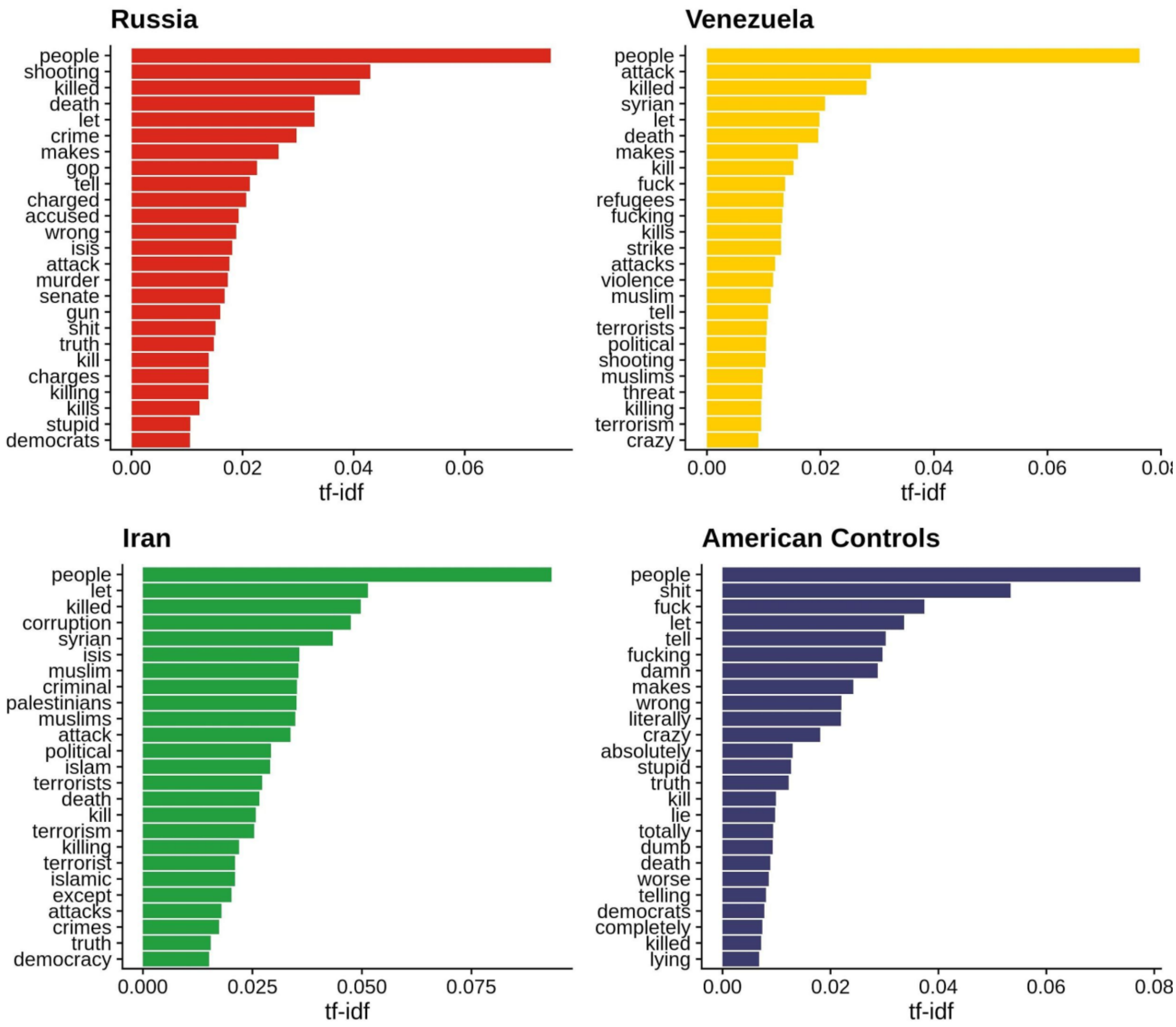

Figure. S1. Term frequency-inverse document frequency of the top 25 polarized words, by Twitter sample. These are the top polarized words in each sample.

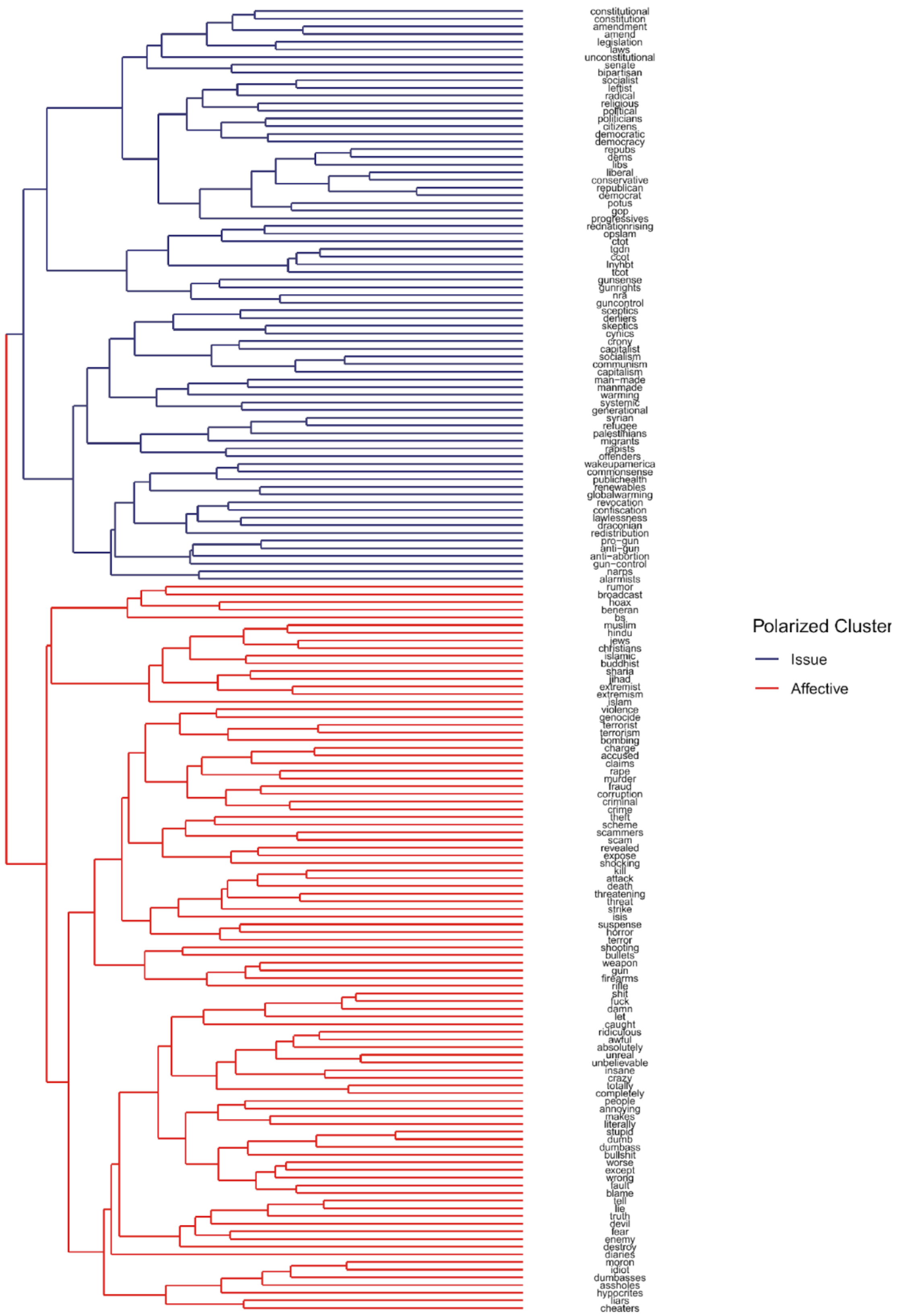

Figure S2. Dendrogram of the hierarchical relationship in the hierarchical clustering analysis, based on 200 dimensions GloVe embeddings.

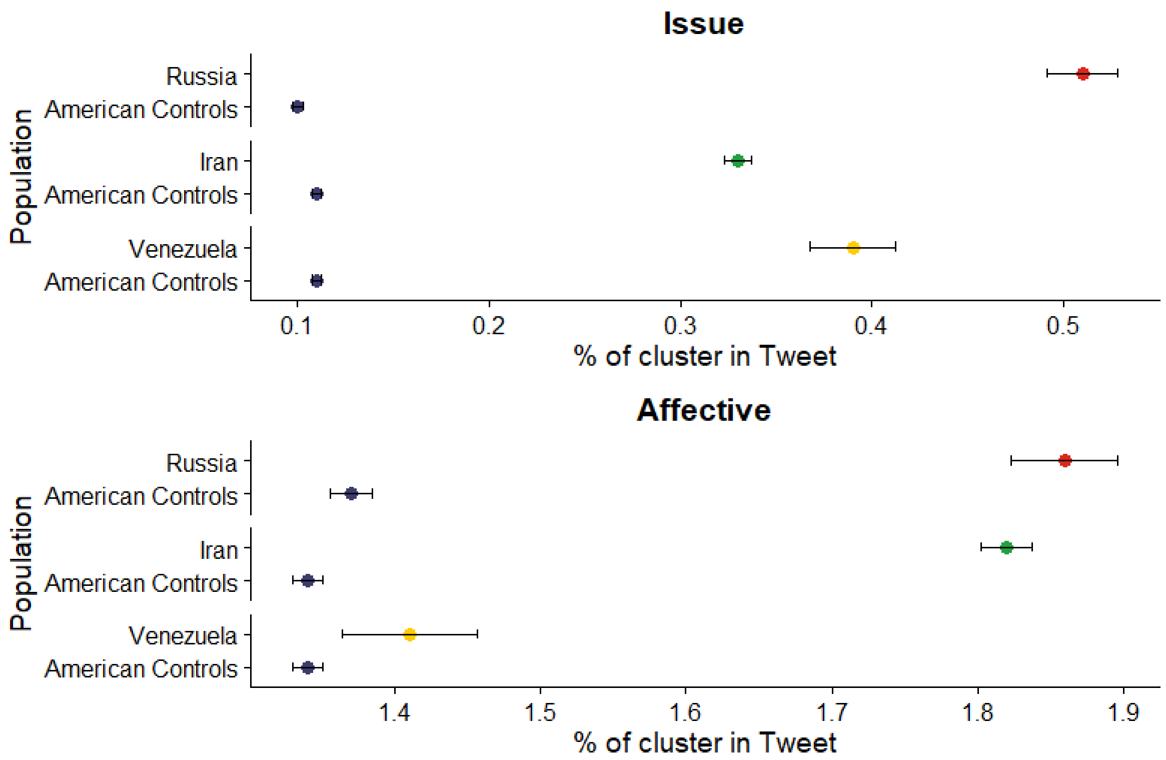

Figure. S3. Polarization score by population (American controls, Russian trolls, Iranian trolls, Venezuelan trolls) and polarization components (Issue and Affective). Points denote means; error bars denote 95% confidence intervals. All comparisons were matched on timeframe.

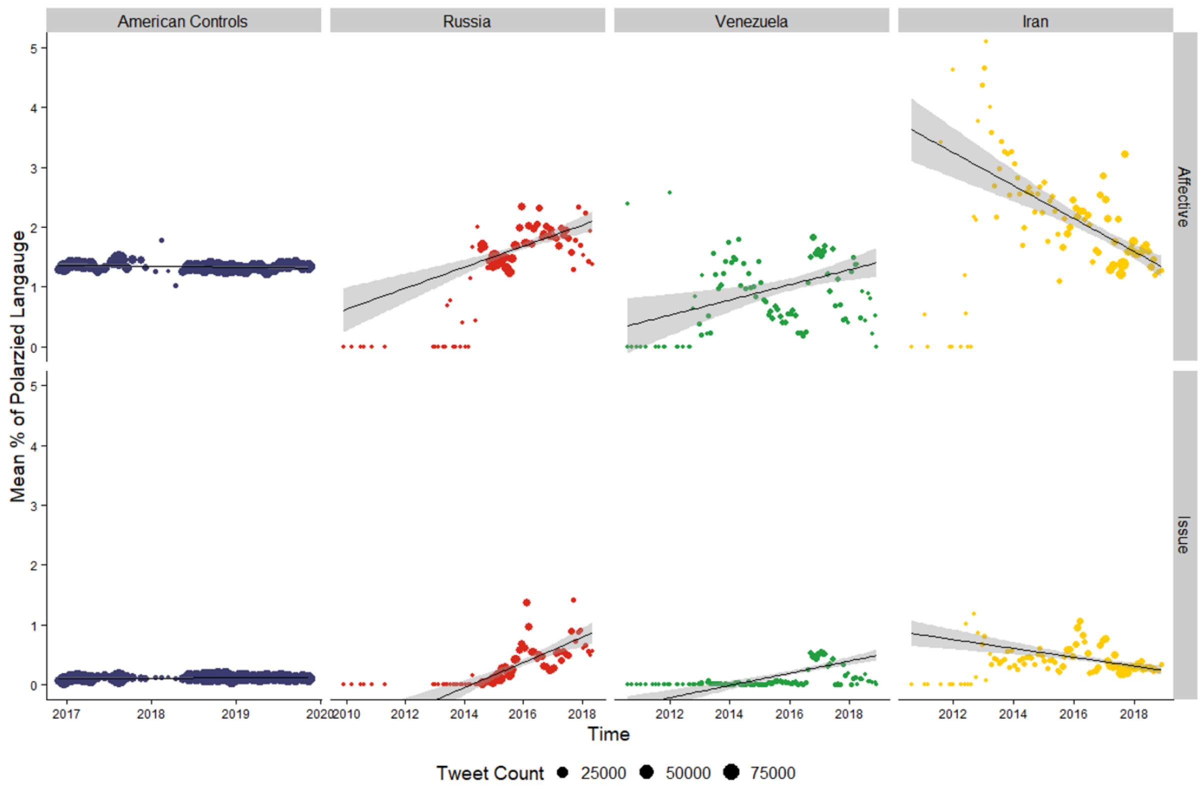

Figure. S4. Scatter plot of the average polarized subcomponent (Affective and Issue) by Twitter sample. Values on the Y-axis represent the average percent of polarized language in the month. Shaded areas around the regression line denote 95% CI. The size of the dots corresponds to the monthly sample size. Note that the Y-axis is fixed to 0-5, data points exceeding this limit are not shown in the figure; the regression lines take these observations into account.

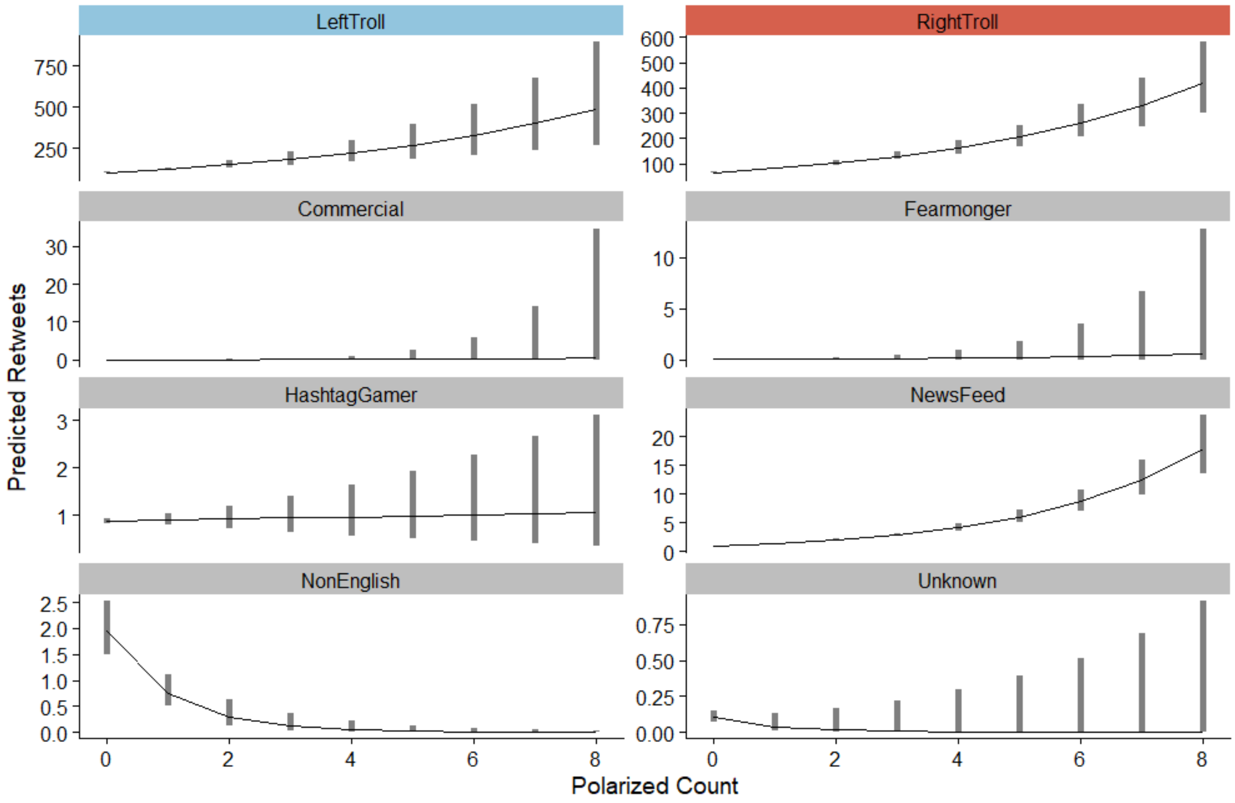

Figure S5. Polarized language predicts retweets in political Russian trolls. The graph depicts the number of retweets predicted for a given tweet as a function of polarized language present in the tweet and type of troll. Bands reflect 95% CIs. For constant Y-axes, see Figure 4.

References

Brady, W. J., Wills, J. A., Jost, J. T., Tucker, J. A., & Van Bavel, J. J. (2017). Emotion shapes the diffusion of moralized content in social networks. *Proceedings of the National Academy of Sciences of the United States of America*, *114*(28), 7313–7318.

Kearney, M. W. (2019). *rreddit*. Github. Retrieved November 3, 2020, from https://github.com/mkearney/rreddit

Pennebaker, J. W., Chung, C. K., Ireland, M., Gonzales, A., & Booth, R. J. (2007). The Development and Psychometric Properties of LIWC2007 (LIWC2007 Manual). *Austin, TX: LIWC*.

Pennington, J., Socher, R., & Manning, C. D. (2014). Glove: Global vectors for word representation. *Proceedings of the 2014 Conference on Empirical Methods in Natural Language Processing (EMNLP)*, 1532–1543.

Schwartz, H. A., Eichstaedt, J. C., Kern, M. L., Dziurzynski, L., Ramones, S. M., Agrawal, M., Shah, A., Kosinski, M., Stillwell, D., Seligman, M. E. P., & Ungar, L. H. (2013). Personality, gender, and age in the language of social media: the open-vocabulary approach. *PloS One*, *8*(9), e73791.

Soliman, A., Hafer, J., & Lemmerich, F. (2019). A Characterization of Political Communities on Reddit. *Proceedings of the 30th ACM Conference on Hypertext and Social Media*, 259–263.

Tien, J. H., Eisenberg, M. C., Cherng, S. T., & Porter, M. A. (2020). Online reactions to the 2017 “Unite the right” rally in Charlottesville: measuring polarization in Twitter networks using media followership. *Applied Network Science*, *5*(1), 10.
